# Systematic review and meta-analysis of the efficacy and safety of amfepramone and mazindol as a monotherapy for the treatment of obese or overweight patients

**DOI:** 10.6061/clinics/2017(05)10

**Published:** 2017-05

**Authors:** Rosa Camila Lucchetta, Bruno Salgado Riveros, Roberto Pontarolo, Rosana Bento Radominski, Michel Fleith Otuki, Fernando Fernandez-Llimos, Cassyano Januário Correr

**Affiliations:** I Laboratório de Serviços Clínicos e Evidências em Saúde, Departamento de Farmácia, Universidade Federal do Paraná (UFPR), Curitiba, PR, BR; II Serviço de Endocrinologia e Metabolismo, Hospital de Clínicas, Universidade Federal do Paraná (UFPR), Curitiba, PR, BR; III Departamento de Ciências Farmacêuticas, Universidade Estadual de Ponta Grossa, Ponta Grossa, PR, BR; IV Research Institute for Medicines (iMed.ULisboa), Department of Social Pharmacy, Faculty of Pharmacy, University of Lisbon, Lisbon, Portugal

**Keywords:** Obesity, Weight loss, Treatment Outcome, Evidence-Based Practice

## Abstract

The aim of this study was to evaluate efficacy and safety of amfepramone, fenproporex and mazindol as a monotherapy for the treatment of obese or overweight patients. A systematic review of primary studies was conducted, followed by a direct meta-analysis (random effect) and mixed treatment comparison. Medline and other databases were searched. Heterogeneity was explored through I^2^ associated with a *p*-value. Of 739 identified publications, 25 were included in the meta-analysis. The global evaluation of Cochrane resulted in 19 studies with a high level of bias and six with unclear risk. Due to the lack of information in primary studies, direct meta-analyses were conducted only for amfepramone and mazindol. Compared to placebo, amfepramone resulted in higher weight loss in the short-term (<180 days; mean difference (MD) -1.281 kg; *p*<0.05; I^2^: 0.0%; *p*=0.379) and long-term (≥180 days; MD -6.518 kg; *p*<0.05; I^2^: 0.0%; *p*=0.719). Only studies with long-term follow up reported efficacy in terms of abdominal circumference and 5-10% weight reduction. These results corroborated the finding that the efficacy of amfepramone is greater than that of placebo. Treatment with mazindol showed greater short-term weight loss than that with placebo (MD -1.721 kg; *p*<0.05; I^2^: 0.9%; *p*=0.388). However, metabolic outcomes were poorly described, preventing a meta-analysis. A mixed treatment comparison corroborated the direct meta-analysis. Considering the high level of risk of bias and the absence of important published outcomes for anti-obesity therapy assessments, this study found that the evaluated drugs showed poor evidence of efficacy in the treatment of overweight and obese patients. Robust safety data were not identified to suggest changes in their regulatory status.

## INTRODUCTION

According to the World Health Organization, approximately 39% of adults were obese or overweight in 2014 1. This population is subject to associated co-morbidities, such as diabetes mellitus type 2, hypertension, coronary heart disease and high risk of death 1–3. Thus, how obesity and its complications burden the health system is readily apparent. The Finkelstein et al. study conducted in the United States found that medical costs in patients with obesity in 2006 were 41.5% higher than that for patients of normal weight. In Brazil, the estimated annual costs for all diseases related to overweight and obese patients are $2.1 billion; approximately 10% of these costs can be attributed to being overweight or obese 4.

Treatment of obese and overweight patients is based on behavioral changes, diet and exercise, with or without pharmacotherapy or bariatric surgery, aiming to lose weight and decrease risk factors 5. Pharmacological options are being studied and have been available in the market since the 1930s. Despite its long history, pharmacotherapy remains the main tenet of scientific and political debate. There is great concern about effectiveness and safety, as many drugs previously available in the market were withdrawn due to the increased incidence of psychiatric and cardiac disorders. Moreover, the addictive effects of drugs, such as rimonabant, fenfluramine and sibutramine, are a major concern 6. Currently, divergences among regulatory agencies are observed. The US Food and Drug Administration (FDA) approved the use of amfepramone (diethypropion), benzphetamine, bupropion + naltrexone, phendimetrazine, phentermine, phentermine + topiramate, liraglutide, lorcaserin and orlistat. The European Medicines Agency (EMA) only recorded bupropion + naltrexone, liraglutide and orlistat. In Brazil, the Agência Nacional de Vigilância Sanitária (Anvisa) approved orlistat, sibutramine, liraglutide and lorcaserin. Moreover, amfepramone is marketed in Mexico, Chile and other countries in Latin America, and mazindol is used in Canada, Japan and several countries in Latin America. For many years, Brazil was mentioned as one of the largest consumers of appetite suppressants in the world, with evidence of irrational use of this drug class 7–10. Therefore, the country was the site of a debate that divided the Anvisa and medical societies over the maintenance record of amfepramone, mazindol and fenproporex. The Chamber of Deputies of Brazil is still discussing an attempt to halt the cancellation of amfepramone, mazindol and fenproporex use, with the justification that the population has few therapeutic options 11. A feasible explanation lies in the low quality of primary and secondary studies that have already been published. Although differences between Anvisa, FDA, EMA and others countries are likely to occur, all organizations are strongly dependent on the quality of randomized clinical trials (RCT) 12,13. Treatment discontinuation, small sample size, low methodological quality and high levels of heterogeneity in meta-analyses are the most common limitations 14–16.

It is therefore important to conduct primary and secondary studies with low risks of bias to serve as cornerstones for political decisions regarding the appropriate regulation of drugs and their use in clinical practice.

Therefore, our study aimed to assess the efficacy and safety of amfepramone, fenproporex and mazindol as a monotherapy for the treatment of obese or overweight patients using a systematic review followed by a meta-analysis.

## METHODS

Standard systematic review and meta-analytic methods were used to conduct and report this analysis 17,18.

### Sources of information and search

A search was conducted in Medline (via PubMed), SCOPUS, Scielo and the Directory of Open Access Journals until March 2016. Manual searches in the references of the included studies were also performed. The terms used were *diethylpropion, amfepramone, diethylpropione, anfepramone, tenuate, femproporex, fenproporex, perphoxene, mazindol, mazindole* and *sanorex* (the search strategy is available in the Supplemental Materials).

The assessed population comprised obese or overweight individuals, taking into account criteria defined by clinical studies, with or without co-morbidities, and without restrictions of age or gender. It excluded studies undertaken in pregnant women; nursing mothers; or patients with hyperthyroidism, pheochromocytoma, glaucoma, prostate adenoma, kidney failure and liver failure. Publications related to congress abstracts, letters, editorials, news and studies that did not exactly report treatment were also excluded.

Primary studies (RCT, cohort, case-control, case report) were included in our systematic review if they assessed the efficacy or safety of amfepramone, fenproporex or mazindol regardless of treatment duration; controlled by placebo, diet, physical activity or another active drug; reporting one or more of weight change, abdominal circumference or frequency of patients who reached 5% or 10% weight loss or metabolic biomarkers; and withdrawal due to adverse reactions (qualitative or quantitative).

### Study selection and risk of bias in each study

Two independent reviewers (BSR and RCL) performed the search and study selection. Data extraction was performed by RCL and fully reviewed by BSR. Disagreements were settled by an opinion of a third researcher (CJC). Only studies reported in Portuguese, English and Spanish were assessed.

The Cochrane tool was adopted to evaluate the quality of the included studies 19.

### Direct meta-analysis and network meta-analysis

Extracted data were organized and analyzed in the Comprehensive Meta-Analysis software for direct meta-analysis, which applied the random-effects model to predict a high level of heterogeneity among studies. Odds ratios (OR) were applied as effect measurements for dichotomous outcomes, whereas mean differences (MD) were used for continuous outcomes. The statistical method for both outcomes was the inverse of variance. The results are presented with 95% confidence intervals (95% CI). Following the Cochrane recommendation, we did not assess publication bias, as none of the meta-analyses included 10 or more studies. Therefore, Egger regression or funnel plot statistical tests were not developed 19. Sensitivity analysis was performed to identify studies likely to increase heterogeneity.

Network meta-analysis using consistency or inconsistency models were built using Addis version 1.16.6 software, (Drugis, Groningen; The Netherlands). A potential scale reduction factor (PSRF) of convergence assessment close to 1.00 (1<PSRF≤1.05) points indicated that convergence was matched among simulations. Bayesian random effect models were used to assess strategies. The results were expressed by means of credibility intervals of 95% (CrI 95%). Effect measurements for continuous outcomes were MD, whereas OR was used for dichotomous outcomes. The placebo was set as the control.

## RESULTS

**Figure 1:**
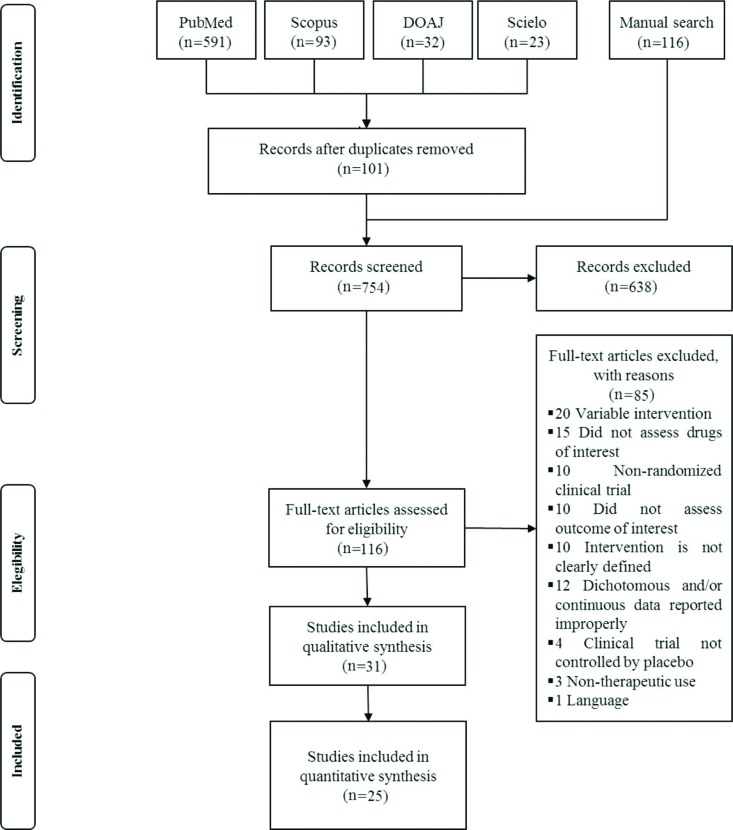
PRISMA flow chart. DOAJ: Directory open access journal.

Our systematic review identified 739 studies published in the assessed databases. Of these, 85 were excluded after full assessment (See Excluded studies in the Supplemental Materials); 31 and 25 were included in our systematic review and the quantitative analysis, respectively (Figure 1).

### Study characteristics

The included studies comprised RCT (n=25) 20–44 and case reports (n=6) 45–50. Cohort and case-control studies that complied with our inclusion criteria were not identified.

Considering only RCTs, the studies were published from 1967 to 2014, mainly in the United States (n=9). Data from 1,965 patients were assessed (median: 50; IIQ 25-75%: 28-80), and 19 studies took into account male and female subjects. However, data from women were more commonly observed (n=965). Twenty-three studies assessed adults, 12 assessed elderly people, 7 assessed adolescents and 2 assessed children. One study did not report age groups. Only 6 studies reported the presence or absence of co-morbidities. Efficacy, safety and metabolic biomarker findings were collected for amfepramone (n=13), mazindol (n=13) and fenproporex (n=1). The most common comparator was placebo (n=25). There were two head-to-head studies comparing d-amphetamine (n=1), mazindol (n=1), fenproporex (n=1) and sibutramine (n=1).

The majority of studies had a follow up of up to 12 weeks (84 days) (median: 84, IIQ 25-75%: 56-84). Two studies applied the methodology of intention-to-treat for assessing results 28,41 (see characteristics of the included studies in our systematic review in the Supplemental Materials).

### Risk of bias in RCT

Considering the Cochrane tool for risk of bias, the global evaluation resulted in 19 studies with high risk of bias and 6 with uncertain risk.

The domains presenting more risk of bias were related to selective reporting (n=9) and other sources of bias (n=11). In the first case, despite no studies reporting any *a priori* project protocol, most studies reported parameters and outcomes of interest in the Methods section, regardless of the lack of results. For the second source of bias, pharmaceutical companies responsible for marketing the drug under assessment were the funding source. Eleven studies mentioned the funding source, whereas 14 did not disclose this information in the conflict of interest statement (see chart of risk of bias in RCT described in the Supplemental Materials).

### Synthesis of Results

#### Direct meta-analysis

##### Efficacy

Only RCTs assessing amfepramone and mazindol compared to placebo were included in the direct meta-analysis.

Treatment with amfepramone led to greater loss of body weight than treatment with placebo in all studies (28,30,37,38,41,44) regardless of daily dosage and treatment duration (MD =-1.291 kg (95% CI: -1.548; -1.035), heterogeneity test *p*=0.000; I^2^: 85.5%). After subgroup analysis of treatment duration (short-term: <180 days and long-term: >180 days) and sensitivity analysis (hypothetic exclusion of Ramos et al. 1964), the result increased to MD=-1.375 kg (95% CI: -1.630; -1.121) and heterogeneity I^2^: 85.5% (*p*=0.000). Nevertheless, homogeneity was identified among studies assessing short-term MD=1.281 kg (95% CI: -1.538; -1.024; *p*=0.379; I^2^: 0.0%). For studies assessing long-term results, MD=-6.518 kg (95% CI: -8.419; -4.617; *p*=0.719; I^2^: 0.0%) (see Summary of findings: efficacy, safety and metabolic outcomes in the Supplemental Materials). Ramos et al. (1964) was identified as the study responsible for a high level of heterogeneity in global analysis. This may have been due to the dosage assessed (25 mg/tid (ter in die) instead of 75 mg/qd (quaque die)) and the methodological quality 37.

The two studies assessing long-term treatment also reported efficacy according to abdominal circumference and 5% and 10% weight reductions, confirming the efficacy of amfepramone over placebo (see summary of findings: efficacy, safety and metabolic outcomes in the Supplemental Materials).

From five studies included in the mazindol meta-analysis, four presented body weight reductions greater than the placebo (MD=-2.396 kg; 95% CI: -3.469; -1.323; I^2^: 77.6%; *p*=0.001) 21,29,32,33,42. In the sensitivity analysis, the result dropped to MD=-1.721 kg after removing Heber et al. 32 (95% CI: -2.164; -1.278; I^2^: 0.9%; *p*=0.388). Heber et al. identified MD strongly favoring mazindol compared to the other studies, without a reasonable explanation for such a difference (see summary of findings: efficacy, safety and metabolic outcomes in the Supplemental Materials).

#### Safety

The treatment included in the safety meta-analysis corresponded to short-term studies only and assessed the following outcomes: individuals presenting one or more adverse reactions, withdrawal due to adverse reactions (see summary of findings: efficacy, safety and metabolic outcomes in the Supplemental Materials) and probability of specific adverse reactions (see summary of findings: adverse reactions in the Supplemental Materials).

Considering the six included studies in the probability of adverse reactions meta-analysis 20,23,24,27,34,35, only one presented results favoring the placebo (52.0% *vs*. 28.8%) 27, with an OR=1.847 (95% CI: 1.057; 3.229); I^2^: 0.0%; *p*=0.619) (see summary of findings: efficacy, safety and metabolic outcomes in the Supplemental Materials). Studies showing greater withdrawal due to adverse reactions in the amfepramone group were not identified. Studies assessing amfepramone reported nausea, dry mouth, constipation, stomach discomfort, dizziness, insomnia, headache, tremor, somnolence, tension and irritation as the most common adverse reactions. Nevertheless, only dry mouth was associated with amfepramone treatment (OR=2.430; 95% CI: 1.248; 4.729; I^2^: 0.0%; *p*=0.651) (see summary of findings: adverse reactions in the Supplemental Materials).

Out of five studies included in the probability of adverse reactions meta-analysis, only one pointed to a greater risk for such outcomes in the mazindol group 31, generating an OR=4.086 (95% CI: 1.780; 9.376; I^2^: 0.0%; *p*=0.53) (see summary of findings: efficacy, safety and metabolic outcomes in the Supplemental Materials). The meta-analysis results showed a higher risk of discontinuation in the mazindol group (OR=2.760; 95% CI: 1.472; 5.175; I^2^: 0.0%; *p*=0.949) (see summary of findings: efficacy, safety and metabolic outcomes in the Supplemental Materials). The most common adverse reactions reported were palpitations, rash, nausea, dry mouth, constipation, bad taste sensation, vomiting, dizziness, insomnia, headache, tension, irritation, dysuria and chills. Only insomnia and constipation were associated with mazindol treatment (OR=8.083; 95% CI: 1.782; 36.674; I^2^: 0.0%; *p*=0.910) and OR=3.906 (95% CI: 1.156; 13.197; I^2^: 0.0%; *p*=0.910), respectively (see summary of findings: adverse reactions in the Supplemental Materials).

Adverse reactions described in the identified case reports for amfepramone were paranoid ideation, restlessness, aphasia, transient ischemic attacks due to vasospasm, schizophrenia, psychotic symptoms, dry mouth, constipation, dizziness and chronic bronchitis 45–48,50, and reactions to fenproporex were withdrawal syndrome, aggression, anxiety, irritability, nightmares and insomnia, followed by severe depression and attempted suicide 49.

#### Metabolic outcomes

It was only possible to carry out meta-analyses for total cholesterol change after mazindol treatment. Despite two studies identifying a decrease in total cholesterol (*p*=0.000) 22,39, our meta-analysis showed high heterogeneity (OR=-21.750 mg/ dL; 95% CI: -39.514; -3.987; I^2^: 95.3%; *p*=0.000), not justified by dosage difference, treatment duration, sample size or study quality (see summary of findings: efficacy, safety and metabolic outcomes in the Supplemental Materials).

In addition to total cholesterol, the following outcomes were also reported: changes in fasting glucose, serum triglycerides, systolic blood pressure, diastolic blood pressure and heart rate. However, meta-analysis was prevented since primary data were reported incorrectly or only qualitatively.

### Network meta-analysis

All evaluations were split into short-term (Table 1) and long-term treatments (Table 2). Outcomes that presented statistical significance were change in body weight, favoring amfepramone and mazindol compared to placebo (Table 1), and discontinuation due to adverse reactions, favoring placebo compared to mazindol (Table 1). Regarding adverse reactions, somnolence and insomnia demonstrated statistical significance. The first favored placebo over amfepramone, and the second favored placebo over mazindol (Table 1). All efficacy outcomes assessing long-term treatment favored amfepramone over placebo (Table 2). Due to a closed circuit of evidence, it was not possible to conduct inconsistency analyses. More information on the network meta-analysis is available in the Supplemental Materials.

## DISCUSSION

### Relevance for decision making

Canada, USA, Japan, Brazil, Mexico, Chile and several countries in Latin America have discussed the regulatory status of anorectics as well as updated their regulations for the registration and trade of these drugs. In an official report in 2007 51, the US FDA required the following criteria for a drug to be registered as an anti-obesity drug: promote statistically significant weight loss compared to placebo in >5% of subjects within one year of treatment or >35% of subjects reaching >5% weight loss. The FDA also requested evidence that new drugs are capable of improving metabolic biomarkers, including blood pressure, lipids and glucose 51. Our review identified only two studies, of 6 and 12 months of follow up, that reported the outcome of >5% weight loss 28,41. The first identified 66.7% of patients reaching such an outcome with amfepramone treatment compared to 25% in the placebo group. The second aforementioned study, assessing amfepramone, fenproporex, mazindol, fluoxetine and sibutramine treatment, identified 71.4%, 69.0%, 72.4%, 34.5% and 73.3%, respectively, of patients reaching body weight loss of >5% compared to 34.4% in the placebo group. These studies also reported metabolic biomarkers in a way that prevented their use in a meta-analysis framework. Among the identified studies assessing short-term treatment, none reported losses of 5% or 10% weight loss, and only two reported data capable of use in meta-analysis, presenting a high level of heterogeneity 22,39. Therefore, according to our systematic review, amfepramone, mazindol and fenproporex do not have enough evidence to comply with the efficacy criteria of the FDA.

Safety parameters, especially related to major adverse reactions, were not identified in clinical trials for the assessed drugs, qualitatively or quantitatively 52. Contributing factors may include the lack of separation of major and minor adverse reactions in primary studies and the small sample size. To corroborate such data, observational studies were searched, identifying only six case reports that included cases of addiction and psychotic disorders. Case report studies often describe unpublished or unknown outcomes of a given treatment 53.

### Importance for research

The systematic review followed by meta-analysis summarized several efficacy and safety results comparing weight loss promoted by pharmacotherapeutic treatment and dieting versus weight reduction only from dieting 54. The main limitations of this analysis reside in the short-term monitoring of clinical trials and the lack of analysis by subpopulations, which limit the use of the results in the clinical population given the heterogeneity of obese and overweight patients. To date, systematic review followed by meta-analysis covering the three drugs evaluated here has not been performed.

The most common efficacy outcome reported in the included studies was mean difference in weight loss between groups. As already discussed, 5% or 10% weight loss was rarely described in RCTs. Jensen et al. recommend an initial target reduction of 5-10% of baseline weight within six months of treatment 5. Metabolic biomarkers were poorly reported, whereas for safety, the risk of withdrawal due to adverse reactions and the description of any adverse event experienced were the most common outcomes. The National Obesity Observatory Standard Evaluation Framework (NOO SEF) 55 recommends that at minimum, weight and height measurements must be reported for the assessment of interventions at three different time points within one year of follow up 55. Another concern is the report of different results during treatment. In most RCTs, the results were not divided across the follow up but instead pooled into one single mean, counteracting NOO SEF recommendations.

The majority of our meta-analysis presented low heterogeneity, which can be explained by the identification bias of heterogeneity, since our meta-analysis comprised few studies and small populations. Hippel (2015) identified that true heterogeneity is more likely to be seen when more studies are included. However, there is not a consensus about the minimum number of studies to carry out a meta-analysis 56,57. Therefore, despite having a small number of meta-analyses with heterogeneity, we cannot exclude the chance of being unable to identify true heterogeneity.

Some RCTs have important limitations that are not sensitive to the Cochrane tool, such as the lack of a thorough description of the population under assessment. Most primary studies assessed obese or overweight adults with or without co-morbidities, reporting pooled results. This practice leads to high heterogeneity and results that are difficult to use in clinical practice.</emph> Such data will hardly clarify decisions for decision makers and clinicians, as results such as these are not representative of specific patient profiles 5.

The risk of bias identified through the Cochrane tool is high, which required us to cautiously interpret the results. According to the Cochrane tool, the most important risk of bias lies in the conflict of interest of researchers, as pharmaceutical companies funded the majority of studies. An observational study of 370 RCTs revealed that funding source, treatment effect and double-blinding are the most relevant predictors of statistically significant results 58.

Other observed limitations were small sample size, low number of head-to-head studies, poor description of therapy adhesion, frequency of major adverse reactions and changes in metabolic biomarker outcomes. Discontinuation of treatment, small sample size, low methodological quality and high level of heterogeneity in the meta-analyses were the most common limitations 14–16.

The limitations of this review include the following: a) The review of gray literature (dissertations, theses, abstracts) was absent. Since the three evaluated drugs have over 50 years of marketing and research, and two of them are no longer marketed in major agencies, there was a low probability of identifying studies of interest. Furthermore, a manual search was performed both in the reference lists of included studies and in official materials of companies and organizations, which resulted in 116 identified publications. b) There was also a language limitation; however, its effect was limited to the exclusion of a study published in Polish. c) With respect to inadequate reporting of dichotomous or continuous data in the primary study, we chose to not contact the authors of the studies to obtain raw data, considering the publication dates extended into past decades (23 of 25 studies were published from 1961 to 1987), and we thus had a low probability of success in contact. d) The effects of the combination of drugs with lifestyle changes, including diet and exercise, were not considered in the analysis; although most studies combined medications with lifestyle changes, they did not report the proposed interventions in detail (e.g., calorie deficit, intensity of physical activity, duration). e) The network meta-analysis was performed containing only placebo as the common comparator, which stems from the lack of clinical trials with head-to-head comparisons.

More studies are needed on the safety of the evaluated drugs, principally based in real practice, to have a stronger basis for recommendations to regulatory bodies or health institutions regarding the anorectic drugs evaluated. Future RCTs should be controlled by active drugs, and the results of these trials should be reported in terms of abdominal circumference, change in body weight, the number of participants who achieved a loss of 5-10% of body weight and any impact on metabolic biomarkers over the course of 3, 6, 9 and 12 months. Some population subgroups have plenty of data in the literature. Thus, they have the potential to be further explored in future assessments as obese or overweight adults without co-morbidities, obese adults with diabetes type II, overweight adults with diabetes type II, obese or overweight adults with hypertension and adolescents and children without co-morbidities.

The majority of identified RCTs present a high risk of bias, mostly related to conflict of interest and selective outcome reporting. Regarding short-term treatment (<180 days), amfepramone and mazindol are more effective than placebo for losing weight. Nevertheless, short-term studies assessing a decrease of 5-10% of body weight were not identified. Mazindol was associated with discontinuation due to adverse reactions. The most common adverse reactions caused by this drug were constipation and insomnia. For amfepramone, dry mouth and somnolence were the most common adverse reactions. Long-term treatments (>180 days) only had results favoring amfepramone (compared to placebo). Regarding safety, associations with adverse reactions or discontinuation due to adverse reactions were not identified. Therefore, considering the high risk of bias and the lack of reporting of important outcomes, such as metabolic biomarkers and loss of at least 5% body weight, this study concludes that amfepramone, mazindol and fenproporex have weak evidence of their effectiveness in the treatment of overweight and obese patients. In addition, robust safety data were not identified, which would allow for suggestions regarding short- and long-term changes in their regulatory status.

## AUTHOR CONTRIBUTIONS

Lucchetta RC, Riveros BS, Pontarolo R, Radominski RB, Otuki MF, Fernandez-Llimos F and Correr CJ contributed substantially to the study conception, development, analysis and/or interpretation of the data and the drafting of the manuscript.

## Figures and Tables

**Table 1:** Network meta-analysis: short-term treatments.

**Efficacy – Mean difference (CrI 95%) – Cut-off =0**				
Change in body weight	AMFEPRAMONE	-	-	
	0.61 (-1.26, 2.27)	MAZINDOL	-	
	**-1.51 (-3.16, -0.32)**	**-2.12 (-3.28, -1.14)**	PLACEBO	
**Safety and tolerability – Odds ratio (CrI 95%) – Cut-off =1**				
Discontinuation due to adverse reactions	AMFEPRAMONE	0.61 (0.12, 3.46)	1.20 (0.44, 2.89)	Participants at least on adverse reaction
	0.51 (0.07, 5.52)	MAZINDOL	1.97 (0.43, 7.38)	
	2.43 (0.36, 19.37)	**4.38 (1.89, 16.38)**	PLACEBO	
**Adverse reactions – Odds ratio (CrI 95%) – Cut-off =1**				
Constipation	AMFEPRAMONE	0.20 (0.01, 2.15)	0.93 (0.24, 2.92)	Nausea
	0.38 (0.02, 5.06)	MAZINDOL	4.48 (0.62, 52.81)	
	1.58 (0.16, 13.41)	4.10 (0.81, 38.94)	PLACEBO	
Dizziness	AMFEPRAMONE	0.00 (0.00, 0.03)	1.20 (0.21, 11.90)	Insomnia
	1.39 (0.23, 12.64)	MAZINDOL	**6.17×10^8^ (48.29, 5.08×10^19^)**	
	2.21 (0.64, 9.22)	1.56 (0.34, 6.18)	PLACEBO	
Headache	AMFEPRAMONE	0.12 (0.00, 1.85)	0.94 (0.28, 3.45)	Irritation
	0.99 (0.14, 5.28)	MAZINDOL	7.57 (0.73, 264.30)	
	0.98 (0.31, 2.29)	1.00 (0.21, 4.57)	PLACEBO	
Dry mouth	AMFEPRAMONE	0.00 (0.00, 0.26)	0.00 (0.00, 0.28)	Vomiting
	0.92 (0.09, 7.69)	MAZINDOL	1.63 (0.06, 77.49)	
	2.77 (0.89, 11.87)	3.04 (0.60, 23.89)	PLACEBO	
Somnolence	AMFEPRAMONE	0.48 (0.04, 4.03)	1.21 (0.37, 3.22)	Tension
	2.09 (0.00, 1132452.48)	MAZINDOL	2.40 (0.37, 21.32)	
	**1240.03 (1.17, 5.63×10^7^)**	402.06 (0.46, 7.63×10^7^)	PLACEBO	

Lower triangle, left: treatment in the column compared to treatment in the row. Upper triangle, right: treatment in the row compared to treatment in the column. Bolded values indicate statistically significant differences.

**Table 2:** Network meta-analysis: long-term treatments.

**Efficacy – Mean difference (CrI 95%) – Cut-off=0**						
Change in body weight	AMFEPRAMONE	-2.09 (-9.21, 4.97)	-3.30 (-9.99, 3.39)	**-6.51 (-11.64, -1.51)**		Change in waist circumference
	-1.99 (-9.13, 4.99)	FENPROPOREX	-1.23 (-8.82, 6.46)	-4.38 (-11.84, 2.63)		
	-2.36 (-9.05, 4.52)	-0.31 (-7.66, 7.24)	MAZINDOL	-3.20 (-10.10, 3.49)		
	**-6.57 (-11.83, -1.36)**	-4.53 (-11.72, 2.46)	-4.16 (-11.06, 2.46)	PLACEBO		
**Efficacy and safety – Odds ratio (CrI 95%) – Cut-off =1**						
10% weight loss	AMFEPRAMONE	1.20 (0.18, 8.15)	0.99 (0.13, 7.00)		**5.56 (1.30, 25.11)**	5% weight loss
	3.64 (0.13, 84.38)	FENPROPOREX	0.84 (0.10, 6.86)		4.56 (0.66, 34.33)	
	3.06 (0.13, 77.72)	0.86 (0.03, 24.94)	MAZINDOL		5.51 (0.79, 40.82)	
	**33.26 (2.73, 577.98)**	9.38 (0.38, 291.18)	10.97 (0.42, 324.51)		PLACEBO	
	AMFEPRAMONE	1.64 (0.20, 19.53)	3.89 (0.31, 150.48)		1.24 (0.24, 6.39)	Discontinuation due to adverse reactions
		FENPROPOREX	2.45 (0.14, 117.99)		0.78 (0.06, 6.97)	
			MAZINDOL		0.33 (0.01, 3.76)	
					PLACEBO	

Lower triangle, left: treatment in the column compared to treatment in the row. Upper triangle, right: treatment in the row compared to treatment in the column. Bolded values indicate statistically significant differences.
