# ERRATUM: Systematic review and meta-analysis of the efficacy and safety of amfepramone and mazindol as a monotherapy for the treatment of obese or overweight patients

**DOI:** 10.6061/clinics/2018/e1err

**Published:** 2018-02-22

## CLINICS 2017;72(05):317-24

In the article **Systematic review and meta-analysis of the efficacy and safety of amfepramone and mazindol as a monotherapy for the treatment of obese or overweight patients**, on page 319, remove the following phrase from the **RESULTS** section / **Synthesis of Results / Direct meta-analysis / Efficacy:**

**“(see the analysis in the Supplemental Materials)”**

