# Supplementary material for: Systematic review and meta-analysis of the efficacy and safety of amfepramone and mazindol as a monotherapy for the treatment of obese or overweight patients
Source: Clinics (Sao Paulo). 2017 May;72(5):317–24. doi: 10.6061/clinics/2017(05)10 (PMC5439101; doi:10.6061/clinics/2017(05)10)
Supplement: Supplementary file 1 [file cln-72-05-317-suppl1.pdf]

Supplemental Materials

Systematic review and meta-analysis of the efficacy and safety of amfepramone and mazindol as a monotherapy for the treatment of obese or overweight patients

Rosa Camila Lucchetta, Bruno Salgado Riveros, Roberto Pontarolo, Rosana Bento Radominski, Michel Fleith Otuki, Fernando Fernandez-Llimos and Cassyano Januário Correr

Clinics (Sao Paulo). 2017 May; 72(5): 317-324. Doi: 10.6061/clinics/2017(05)10.

Search strategy used for the systematic review of primary studies

<u>MEDLINE (via PUBMED)</u>

((diethylpropion[tiab] OR amfepramone[tiab] OR diethylpropione[tiab] OR anfepramone[tiab] OR tenuate[tiab] OR diethylpropion[mesh] OR femproporex[tiab] OR fenproporex[tiab] OR perphoxene[tiab] OR mazindol[tiab] OR mazindole[tiab] OR sanorex[tiab] OR mazindol[mesh]) NOT (letter[pt] OR editorial[pt] OR review[pt]) NOT (“guinea pig”[tiab] OR rat[tiab] OR rats[tiab] OR mouse*[tiab] OR mice*[tiab] OR monkey*[tiab] OR hamster*[tiab] OR animal*[tiab] OR “in vitro”[tiab]))

<u>SCOPUS (ADVANCED SEARCH)</u>

TITLE-ABS-KEY(“diethylpropion” OR “amfepramone” OR “diethylpropione” OR “anfepramone” OR “tenuate” OR “femproporex” OR “fenproporex” OR “perphoxene” OR “mazindol” OR “mazindole” OR “sanorex”) NOT DOCTYPE(“ed” OR “le” OR “re”) NOT TITLE-ABS-KEY(“guinea pig” OR “rat” OR “rats” OR “mouse” OR “mice” OR “monkeys” OR “hamster” OR “animal” OR “in vitro”)

Editorial-ed/Letter-le/Review-re

<u>DIRECTORY OF OPEN ACCESS JOURNALS</u>

SEARCH ALL

“diethylpropion” OR “amfepramone” OR “diethylpropione” OR “anfepramone” OR “tenuate” OR “femproporex” OR “fenproporex” OR “perphoxene” OR “mazindol” OR “mazindole” OR “sanorex”

SCIELO

ALL INDEXES; REGIONAL

“diethylpropion” OR “amfepramone” OR “diethylpropione” OR “anfepramone” OR “tenuate” OR “femproporex” OR “fenproporex” OR “perphoxene” OR “mazindol” OR “mazindole” OR “sanorex”

Primary studies excluded from the systematic review

Variable intervention (n = 20)

1. Chiorboli E, Scazulca A. Emprego clínico de nova forma farmacêutica do femproporex no tratamento da hiperorexia em obesidade exógena. Folha Med. 1975;71(4):423–30.
2. Decina L, Tanyol H. Treatment of obesity with a new anorexiant, diethylpropion, without special stress on diet. N Y State J Med. 1960/09/01 ed. 1960;60:2702–5.
3. Hagiwara M, Tsuchida A, Hyakkoku M, Nishizato K, Asai T, Nozawa Y, et al. Delayed onset of pulmonary hypertension associated with an appetite suppressant, mazindol: a case report. Jpn Circ J. 2000 Mar;64(3):218–21.
4. Hendon JR, Urbach S. Use of diethylpropion in obese diabetic patients. Metabolism. 1962/03/01 ed. 1962;11:337–41.
5. Horie NC, Cercato C, Mancini MC, Halpern A. Long-term pharmacotherapy for obesity in elderly patients: a retrospective evaluation of medical records from a specialized obesity outpatient clinic. Drugs Aging. 2010 Jun 1;27(6):497–506.
6. Inoue S. Clinical studies with mazindol. Obes Res. 1995 Nov;3 Suppl 4:549S – 552S.
7. Ishikawa T, Nakaya N, Nakamura H, Goto Y. Effect of an anorectic agent, AN 448 on weight reduction and plasma lipids. Keio J Med. 1975 Jul;24(3):211–8.
8. Matthews PA. Diethylpropion in the treatment of obese patients seen in general practice. Curr Ther Res Clin Exp. 1975/04/01 ed. 1975;17(4):340–6.
9. McEwen J, Meyboom RH. Testicular pain caused by mazindol. Br Med J (Clin Res Ed). 1983/12/10 ed. 1983 Dec 10;287(6407):1763–4.
10. Mortes J. Contribution to a study of a new lipolytic and anorectic drug: fenproporex. Folia Clin Int. 1973/02/01 ed. 1973;23(2):134–51.
11. Nash J. The treatment of obesity by control of appetite with diethylproprion (Tenuate). J Ir Med Assoc. 1961/01/01 ed. 1961;48:15–7.
12. Nishikawa T, Iizuka T, Omura M, Kuramoto N, Miki T, Ito H, et al. Effect of mazindol on body weight and insulin sensitivity in severely obese patients after a very-low-calorie diet therapy. Endocr J. 1996 Dec;43(6):671–7.
13. Perlstein IB. Diethylpropion in the clinical management of obese patients. Appl Ther. 1962 Dec;4:1110–25 passim.
14. Rihmer Z, Révai K, Arató M, Perényi A. Two case reports of mazindol-induced

depression. Am J Psychiatry. 1984 Nov;141(11):1497–8.

15. Rosenberg BA. A double-blind study of diethylpropion in obesity. Am J Med Sci. 1961/08/01 ed. 1961;242:201–6.
16. Russek HI. Control of obesity in patients with angina pectoris: a double-blind study with diethylpropion hydrochloride. Am J Med Sci. 1966 Apr;251(4):461–4.
17. Ryan DH, Johnson WD, Myers VH, Prather TL, McGlone MM, Rood J, et al. Nonsurgical weight loss for extreme obesity in primary care settings: results of the Louisiana Obese Subjects Study. Arch Intern Med. 2010/01/27 ed. 2010 Jan 25;170(2):146–54.
18. Sanders M, Breidahl H. The effect of an anorectic agent (Mazindol) on control of obese diabetics. Med J Aust. 1976/10/09 ed. 1976 Oct 9;2(15):576–7.
19. Seedat YK, Reddy J. Letter: Diethylpropion hydrochlorida (Tenuate Dospan) in the treatment of obese hypertensive patients. S Afr Med J. 1974/03/23 ed. 1974 Mar 23;48(14):569.
20. Zaragoza R, López M, Villanueva S, Ortiz R, Villanueva G. Eficacia y seguridad de dos formulaciones de fenproporex de liberación lenta como tratamiento de obesidad. Rev Mex. 2005;16(4):146–54.

Did not assess drugs of interest (n = 15)

1. Abenhaim L, Moride Y, Brenot F, Rich S, Benichou J, Kurz X, et al. Appetite-suppressant drugs and the risk of primary pulmonary hypertension. N Engl J Med. 1996;335(9):609–16.
2. Altschuler S, Conte A, Sebok M, Marlin RL, Winick C. Three controlled trials of weight loss with phenylpropanolamine. Int J Obes. 1982 Jan;6(6):549–56.
3. Andersen T, Hyldstrup L, Quaade F. Formula diet in the treatment of moderate obesity. Int J Obes. 1983/01/01 ed. 1983 Jan;7(5):423–30.
4. Follath F, Burkart F, Schweizer W. Drug-induced pulmonary hypertension? Br Med J. 1971;1:265–6.
5. Kramer M, Lane D. Aminorex, dexfenfluramine, and primary pulmonary hypertension. J Clin Epidemiol. 1998;51(4):361–4.
6. Naeije R, De Backer D, Vachiéry JL, De Vuyst P. High-altitude pulmonary edema with primary pulmonary hypertension. Chest. 1996/07/01 ed. 1996 Jul;110(1):286–9.
7. O'Keefe JC, Butrous GS, Dymond DS, Littlejohns P, Peters N, Banim SO. Ventricular arrhythmias complicating weight reduction therapy in a patient with a prolonged QT

interval. Postgr Med J. 1985/05/01 ed. 1985 May;61(715):419–21.

8. Ost LG, Gotestam L. Behavioral and pharmacological treatments for obesity: an experimental comparison. Addict Behav. 1976;1:331–8.
9. Quaade F, Hyldstrup L, Andersen T. The Copenhagen PRODI project: preliminary results. Int J Obes. 1981 Jan;5(3):263–6.
10. Resnick M, Joubert L. A Double-blind evaluation of an anorexiant, a placebo and diet alone in obese subjects. Canad Med Ass J. 1967;97:1011–5.
11. Stuart R. Behavioral control of overeating. Obes Res. 1967;4(4):411–7.
12. Stunkard A, LW C, O'Brien R. Controlled trial of behaviour therapy, pharmacotherapy, and their combination in the treatment of obesity. Lancet. 1980;1045–7.
13. Thomas SH, Butt AY, Corris PA, Egan JJ, Higenbottam TW, Madden BP, et al. Appetite suppressants and primary pulmonary hypertension in the United Kingdom. Br Heart J. 1995/12/01 ed. 1995 Dec;74(6):660–3.
14. Unger P, Nortier J, Muniz Martinez M-C, Plein D, Vandenbossche J-L, Vereerstraeten P, et al. High prevalence of fenfluramine-related aortic regurgitation in women with end-stage renal disease secondary to Chinese herb nephropathy. Nephrol Dial Transplant. 2003/04/11 ed. 2003 May;18(5):906–10.
15. Wadden TA, Berkowitz RI, Womble LG, Sarwer DB, Phelan S, Cato RK, et al. Randomized trial of lifestyle modification and pharmacotherapy for obesity. N Engl J Med. 2005 Nov 17;353(20):2111–20.

Non-randomized clinical trials (n = 10)

1. Follows O. A comparative trial of fenfluramine and diethylpropion in obese, hypertensive patients. Br J Clin Pract. 1971;25(5):236–8.
2. Garcia De Los Rios M, Bahamondes G, Valiente S, Vallejos J, Canessa I. [Anorexigenic action of diethylpropion. Preliminary study]. Rev Med Chil. 1961/08/01 ed. 1961;89:595–7.
3. Gilbert S, Garrow JS. A prospective controlled trial of outpatient treatment for obesity. Hum Nutr Clin Nutr. 1983 Jan;37(1):21–9.
4. Glazer N. A Double-blind evaluation of a weight reduction programme in private practice. J Int Med Res. 1975;3:436–40.
5. Hadler A. Studies of aminorex, a new anorexigenic agent. Joruanl Clin Pharmacol. 1967;296–302.
6. Le Riche WH, Csima A. A long-acting appetite suppressant drug studied for 24 weeks in

both continuous and sequential administration. Can Med Assoc J. 1967 Oct 21;97(17):1016–20.

7. Malchow-Moller A, Larsen S, Hey H, Stokholm KH, Juhl E, Quaade F, et al. Ephedrine as an anorectic: the story of the "Elsinore pill." Int J Obes. 1981/01/01 ed. 1981 Jan;5(2):183–7.
8. McKay RH. Long-term use of diethylpropion in obesity. Curr Med Res Opin. 1973 Jan;1(8):489–93.
9. Rath R, Vondra K, Bass A, Kujalova V, Wenkeova J. The effect of mazindol on metabolic and regulatory changes in obese women during weight reduction. Int J Obes. 1979/01/01 ed. 1979 Jan;3(2):133–40.
10. Sonka J, Límanová Z, Zbirková A, Kratochvíl O. Effects of diet, exercise and anorexigenic drugs on serum thyroid hormones. Endokrinologie. 1980/12/01 ed. 1980 Dec;76(3):351–6.

Did not assess outcomes of interest (n = 10)

1. Alfaro RD, Gracanin V, Schlueter EA. A clinical pharmacologic evaluation of diethylpropion. J Lancet. 1960/11/01 ed. 1960;80:526–30.
2. Chait LD, Uhlenhuth EH, Johanson CE. The discriminative stimulus and subjective effects of phenylpropanolamine, mazindol and d-amphetamine in humans. Pharmacol Biochem Behav. 1986/06/01 ed. 1986 Jun;24(6):1665–72.
3. Dolecek R. Endocrine studies with mazindol in obese patients. Pharmatherapeutica. 1980/01/01 ed. 1980 Jan;2(5):309–16.
4. Griggs RC, Moxley 3rd RT, Mendell JR, Fenichel GM, Brooke MH, Miller PJ, et al. Randomized, double-blind trial of mazindol in Duchenne dystrophy. Muscle Nerve. 1990/12/01 ed. 1990;13(12):1169–73.
5. Jasinski DR, Nutt JG, Griffith JD. Effects of diethylpropion and d-amphetamine after subcutaneous and oral administration. Clin Pharmacol Ther. 1974 Oct;16(4):645–52.
6. Jonsson CO, Sjoberg L. Studies in the psychological effects of a new drug (diethylpropion). Time curves for five subjective variables. Scand J Psychol. 1967/01/01 ed. 1967;8(1):39–46.
7. Porikos KP, Sullivan AC, Mc Ghee B, Van Itallie TB. An experimental model for assessing effects of anorectics on spontaneous food intake of obese subjects. Clin Pharmacol Ther. 1980/06/01 ed. 1980 Jun;27(6):815–22.
8. Rosse RB, Johri SK, Deutsch SI. Pupillary changes associated with the development of

stimulant-induced mania: a case report. Clin Neuropharmacol. 1997/06/01 ed. 1997 Jun;20(3):270–5.

9. Sjoberg L, Jonsson CO. Studies in the psychological effects of a new drug (diethylpropion): individual differences. Scand J Psychol. 1967/01/01 ed. 1967;8(2):81–7.
10. Stokholm KH, Hansen MS. Lowering of serum total T3 during a conventional slimming regime. Int J Obes. 1983/01/01 ed. 1983 Jan;7(3):195–9.

The intervention was not clearly defined (n = 10)

1. Atkinson RL, Greenway FL, Bray GA, Dahms WT, Molitch ME, Hamilton K, et al. Treatment of obesity: comparison of physician and nonphysician therapists using placebo and anorectic drugs in a double-blind trial. Int J Obes. 1977 Jan;1(2):113–20.
2. Bertol V, Ara JR, Oliveros A, Gutiérrez AI. Subarachnoid hemorrhage caused by fenproporex consumption. Neurologia. 1991;6(7):268–9.
3. Bridgman JF, Buckler JM. Letter: Drug-induced gynaecomastia. Br Med J. 1974 Aug 24;3(5929):520–1.
4. Brooke D, Kerwin R, Lloyd K. Diethylpropion hydrochloride-induced psychosis. Br J Psychiatry. 1988 Apr;152:572–3.
5. Carney MW. Diethylpropion and psychosis. Clin Neuropharmacol. 1988/04/01 ed. 1988 Apr;11(2):183–8.
6. Harrison LC, King-Roach AP, Sandy KC. Effects of mazindol on carbohydrate and insulin metabolism in obesity. Metabolism. 1975/12/01 ed. 1975 Dec;24(12):1353–65.
7. Inoue S, Egawa M, Satoh S, Saito M, Suzuki H, Kumahara Y, et al. Clinical and basic aspects of an anorexiant, mazindol, as an antiobesity agent in Japan. Am J Clin Nutr. 1992 Jan;55(1 Suppl):199S – 202S.
8. Mathewson FA. Changes in the electrocardiogram following the ingestion of diethylpropion. Can Med Assoc J. 1960/06/11 ed. 1960 Jun 11;82(24):1227–8.
9. Noble RE. A controlled study of a weight reduction regimen. Curr Ther Res Clin Exp. 1971 Nov;13(11):685–91.
10. Schneeberg NG. Clinical evaluation of diethylpropion (Tenuate), a new anorectic agent. J Albert Einstein Med Cent. 1961/07/01 ed. 1961;9:191–7.

Dichotomous and/or continuous data were reported improperly (n = 12)

1. Abramson R, Garg M, Cioffari A, Rotman PA. An evaluation of behavioral techniques reinforced with an anorectic drug in a double-blind weight loss study. J Clin Psychiatry.

1980 Jul;41(7):234–7.

2. Cunningham GLW. Diethylpropion in the treatment of obesity. J Coll Gen Pract. 1963/05/01 ed. 1963 May;6:347–9.
3. Hadden DR, Lucey C. Diethylpropion in the treatment of obesity. A cross-over trial of a long-acting preparation. Ulster Med J. 1961/12/01 ed. 1961;30:109–13.
4. Haugen HN. Double blind cross-over study of a new appetite suppressant AN 448. Eur J Clin Pharmacol. 1975/01/01 ed. 1975;8(1):71–4.
5. Johnson W, Hughes J. Mazindol: its efficacy and mode of action in generating weight loss. Addict Behav. 1979/01/01 ed. 1979;4(3):237–44.
6. Le Riche WH, Csima A. A long-acting appetite suppressant drug studied for 24 weeks in both continuous and sequential administration. Can Med Assoc J. 1967 Oct 21;97(17):1016–20.
7. Miach PJ, Thomson W, Doyle AE, Louis WJ. Double-blind cross-over evaluation of mazindol in the treatment of obese hypertensive patients. Med J Aust. 1976/09/04 ed. 1976;2(10):378–80.
8. Rodin J, Elias M, Silberstein LR, Wagner A. Combined behavioral and pharmacologic treatment for obesity: predictors of successful weight maintenance. J Consult Clin Psychol. 1988 Jun;56(3):399–404.
9. Schwartz L. A non-amphetamine anorectic agent: preclinical background and a double-blind clinical trial. 1975. 3:328–32.
10. Silverstone JT, Solomon T. The long-term management of obesity in general practice. Br J Clin Pract. 1965 Jul;19:395–8.
11. Silverstone T. Intermittent treatment with anorectic drugs. Practitioner. 1974 Aug;213(1274):245–52.
12. Walker BR, Ballard IM, Gold JA. A multicentre study comparing mazindol and placebo in obese patients. J Int Med Res. 1977 Jan;5(2):85–90.

The clinical trial was not controlled with a placebo (n = 4)

1. Hernandez A, Saavedra M. Tratamiento a corto plazo de la obesidad: compracion entre anorexigeno y modificacion conductual. Rev Med Chile. 1984;112:132–8.
2. Murphy JE, Donald JF, Molla AL, Crowder D. A comparison of mazindol (Teronac) with diethylpropion in the treatment of exogenous obesity. J Int Med Res. 1975 Jan;3(3):202–6.
3. Silverstone JT, Cooper RM, Begg RR. A comparative trial of fenfluramine and

diethylpropion in obesity. Br J Clin Pract. 1970 Oct;24(10):423–5.<br>4. Valle-Jones JC, Brodie NH, O'Hara H, O'Hara J, McGhie RL, Vallé-Jones JC. A comparative study of phentermine and diethylpropion in the treatment of obese patients in general practice. Pharmatherapeutica. 1983/01/01 ed. 1983 Jan;3(5):300–4.
**Non-therapeutic use (n = 3)**
1. Caplan J, Ch B. Habituation to Diethyipropion (Tenuate) Rapid Laboratory Diagnosis of an Outbreak of Influenza. Can Med Assoc J. 1963;88:943–4.<br>2. Whitlock FA, Nadorfi MI. Diethylpropion and psychosis. Med J Aust. 1970 Dec 5;2(23):1097.<br>3. Wright GJ, Lang JF, Lemieux RE, Goodfriend Jr. MJ. The objective and timing of drug disposition studies, appendix III. Diethylpropion and its metabolites in the blood plasma of the human after subcutaneous and oral administration. Drug Metab Rev. 1975/01/11 ed. 1975;4(2):267–76.
**Language (n = 1)**
1. Sicinski A, Stasiakowa L. [Mazindol in the treatment of simple obesity]. Pol Arch Med Wewn. 1976/06/01 ed. 1976;55(6):581–7.

Brief of risk of bias assessment of randomized clinical trials

	A	B	C	D	E	F	G	H	Funding source
Andelman 1967 (1)	+	-	+	?	?	-	?	-	NR
Baird 1977 (2)	?	?	?	?	?	?	-	-	Eaton Laboratories
Bandisode 1975 (3)	?	?	+	?	?	-	?	-	NR
Bolding 1968 (4)	+	+	+	+	?	-	?	-	NR
Bolding 1974 (5)	?	?	+	?	+	-	?	-	NR
Bradley 1974 (6)	?	+	+	?	?	+	?	?	NR
Campagnoli 1976 (7)	+	?	?	?	?	+	?	?	NR
Carney 1975 (8)	?	?	?	?	+	?	-	-	Merrell-National Laboratories, Ltd.
Cercato 2009 (9)	+	-	+	?	+	?	-	-	Medley Pharmaceutical
DeFelice 1973 (10)	?	?	+	?	-	-	-	-	Sandoz-Wander, Inc.
Elliot 1978 (11)	?	?	?	?	+	?	-	-	Merrell-National Laboratories, Ltd.
Hadler 1972 (12)	?	+	?	+	-	?	?	-	NR
Heber 1975 (13)	?	?	?	+	-	+	-	-	Sandoz-Wander, Inc.
Maclay 1977 (14)	?	?	?	?	-	-	?	-	NR
McQuarrie 1975 (15)	?	?	+	?	?	-	?	-	NR
Nolan 1975 (16)	?	?	+	?	+	?	?	?	NR
Parsons 1981 (17)	?	?	+	?	+	+	?	?	NR
Ramos 1964 (18)	?	?	?	?	?	+	-	-	Merrell-National Laboratories, Ltd.
Seaton 1961 (19)	?	+	+	+	+	+	-	-	Merrell-National Laboratories, Ltd.
Slama 1978 (20)	?	?	?	?	?	-	?	-	NR
Smith 1975 (21)	?	?	+	?	-	?	-	-	Wander Pharmaceuticals
Suplicy 2014 (22)	?	?	-	?	+	?	-	-	Medley Pharm., Aché Pharm. and Apparenza
Thorpe 1975 (23)	?	?	?	?	+	+	?	?	NR
Wallace 1976 (24)	?	?	?	?	-	-	-	-	Sandoz-Wander, Inc.
Williams 1968 (25)	+	?	+	?	+	+	?	?	NR

NR: not reported. (+): low risk of bias; (?): unclear risk; (-): high risk of bias. **A** – random sequence generation (selection bias), **B** - allocation concealment (selection bias), **C** - blinding of participants and personnel (performance bias), **D** - blinding of outcome assessment (detection bias), **E** - incomplete outcome data (attrition bias), **F** - selective reporting (reporting bias), **G** - other sources of bias, **H** - Cochrane risk of bias.

References:

1. Andelman MB, Jones C, Nathan S. Treatment of obesity in underprivileged adolescents. Comparison of diethylpropion hydrochloride with placebo in a double-blind study. Clin Pediatr (Phila). 1967; 6(6):327–30.
2. Baird IM, Howard AN. A double-blind trial of mazindol using a very low calorie formula diet. Int J Obes. 1977; 1(3):271–8.

3. Bandisode MS, Boshell BR. Double-blind clinical evaluation of mazindol (42-548) in obese diabetics. Curr Ther Res Clin Exp. 1975; 18(6):816–24.
4. Bolding OT. A double-blind evaluation of Tenuate dospan in overweight patients from a private gynecologic practice. J Med Assoc State Ala. 1968; 38(3):209–12.
5. Bolding OT. Diethylpropion hydrochloride: an effective appetite suppressant. Curr Ther Res Clin Exp. 1974; 16(1):40–8.
6. Bradley H, Blum N, Scheib R. Mazindol in obesity with known cardiac disease. J Int Med Res. 1974; 2:347.
7. Campagnoli M. [Clinical trial with a nonamphetaminic anoretic]. Rev ibérica Endocrinol. 1976/09/01. 1976; 23(137):463–83.
8. Carney DE, Tweddell ED. Double blind evaluation of long acting diethylpropion hydrochloride in obese patients from a general practice. Med J Aust. 1975; 1(1):13–5.
9. Cercato C, Roizenblatt V a, Leança CC, Segal A, Lopes Filho a P, Mancini MC, et al. A randomized double-blind placebo-controlled study of the long-term efficacy and safety of diethylpropion in the treatment of obese subjects. Int J Obes (Lond). 2009; 33(8):857–65.
10. Defelice EA, Chaykin LB, Cohen A. Double-blind clinical evaluation of mazindol, dextroamphetamine, and placebo in treatment of exogenous obesity. Curr Ther Res Clin Exp. 1973; 15(7):358–66.
11. Elliott BJ. A double-blind controlled study of the use of diethylpropion hydrochloride (Tenuate) in obese patients in a rural practice. N Z Med J. 1978; 88(622):321–2.
12. Hadler AJ. Mazindol, a new non-amphetamine anorexigenic agent. J Clin Pharmacol New Drugs. 1972/11/01. 1972; 12(11):453–8.
13. Heber KR. Double-blind trial of mazindol in overweight patients. Med J Aust. 1975; 2(14):566–7.
14. Maclay WP, Wallace MG. A multi-centre general practice trial of mazindol in the treatment of obesity. Practitioner. 1977; 218(1305):431–4.
15. McQuarrie HG. Clinical assessment of the use of an anorectic drug in a total weight reduction program. Curr Ther Res Clin Exp. 1975; 17(5):437–43.
16. Nolan GR. Use of an anorexic drug in a total weight reduction program in private practice. Curr Ther Res Clin Exp. 1975/08/01. 1975; 18(2):332–7.
17. Parsons WB, Parsons Jr. WB. Controlled-release diethylpropion hydrochloride used in a program for weight reduction. Clin Ther. 1981/01/01. 1981; 3(5):329–35.
18. Ramos EC de. The use of diethylpropion in the treatment of obesity. Br J Clin Pract. 1964; 18:210–1.
19. Seaton DA, Duncan LJ, Rose K, Scott AM. Diethyl-propion in the treatment of “refractory” obesity. Br Med J. 1961/04/08. 1961; 1(5231):1009–11.
20. Slama G, Selmi A, Hautecouverture M, Tchobroutsky G. Double blind clinical trial of mazindol on weight loss blood glucose, plasma insulin and serum lipids in overweight diabetic patients. Diabète & métabolisme. 1978; 4(3):193–9.
21. Smith RG, Innes JA, Munro JF. Double-blind evaluation of mazindol in refractory obesity. BMJ. 1975; 3(5978):284–284.
22. Suplicy H, Boguszewski CL, Santos CMC Dos, Desterro de Figueiredo M do, Cunha DR, Radominski R. A comparative study of five centrally acting drugs on the pharmacological treatment of obesity. Int J Obes (Lond). 2013/11/30. Nature Publishing Group; 2014; 38(8):1097–103.
23. Thorpe PC, Isaac PF, Rodgers J. A controlled trial of mazindol (Sanjorex, Teronac) in the management of the obese rheumatic patients. Curr Ther Res Clin Exp. 1975; 17(2):149–55.
24. Wallace AG. AN 448 Sandoz (Mazindol) in the treatment of obesity. Med J Aust.

1976; 1(11):343–5.
25. Williams J. Trial of a long-acting preparation of diethylpropion in obese diabetics. Practitioner. 1968; 200(197):411–4.

Risk of bias in the included randomized clinical trials

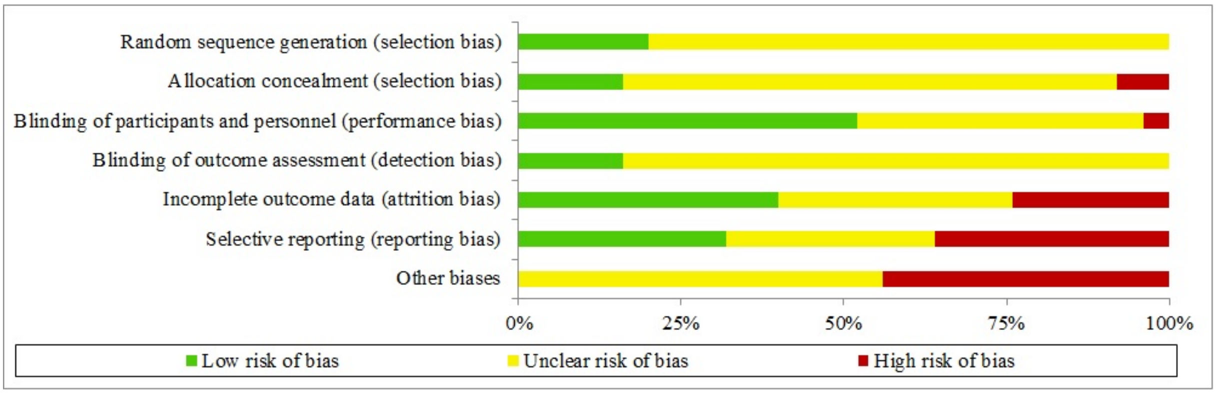

Network meta-analysis: Outcomes, included studies and number of participants and studies

Outcomes	Included studies	# Participants (# studies)
Short-term treatments (alternatives: anfepramone, mazindol, and placebo)		
Dry mouth	Andelman 1967, Bolding 1968, Bradley 1974, Campagnoli 1976, Carney 1975, Cercato 2009, Hadler 1972, Thorpe 1975	485 (8)
Headache	Andelman 1967, Bandisode 1975, Bolding 1968, Bolding 1974, Campagnoli 1976, Carney 1975, Cercato 2009, Hadler 1972, Heber 1975, McQuarrie 1975, Nolan 1975, Thorpe 1975	683 (12)
Constipation	Bandisode 1975, Bolding 1968, Carney 1975, Cercato 2009, Hadler 1972, Heber 1975, Thorpe 1975	423 (7)
Discontinuation due to adverse reactions	Bandisode 1975, Carney 1975, Cercato 2009, DeFelice 1973, Hadler 1972, Heber 1975, Maclay 1977, Parsons 1981, Slama 1978, Smith 1975, Thorpe 1975, Wallace 1976, Williams 1968	1082 (13)
Insomnia	Andelman 1967, Bandisode 1975, Bolding 1968, Bolding 1974, Carney 1975, Hadler 1972, Heber 1975, Thorpe 1975	495 (8)
Irritation	Andelman 1967, Bolding 1968, Cercato 2009, Heber 1975, Thorpe 1975	294 (5)
Change in body weight	Baird 1977, DeFelice 1973, Eliot 1978, Heber 1975, Maclay 1977, Seaton 1961, Thorpe 1975, Williams 1968	711 (8)
Nausea	Andelman 1967, Bandisode 1975, Bolding 1968, Bolding 1974, Carney 1975, Hadler 1972, McQuarrie 1975, Thorpe 1975	486 (8)

Participants with at least one adverse reaction	Andelman 1967, Bolding 1968, Bolding 1974, Bradley 1974, Carney 1975, Hadler 1972, McQuarrie 1975, Nolan 1975, Thorpe 1975	499 (9)
Somnolence	Andelman 1967, Bandisode 1975, Bolding 1974	205 (3)
Tension	Andelman 1967, Bandisode 1975, Bolding 1968, Heber 1975, McQuarrie 1975, Nolan 1975	342 (6)
Dizziness	Andelman 1967, Bandisode 1975, Bolding 1968, Bolding 1974, Bradley 1974, Campagnoli 1976, Carney 1975, Cercato 2009, Hadler 1972, Heber 1975, Thorpe 1975, Williams 1968	733 (12)
Vomiting	Hadler 1972, Heber 1975, McQuarrie 1975	151 (3)
Long-term treatments (alternatives: anfepramone, fenproporex, mazindol, and placebo)		
10% weight loss	Cercato 2009, Suplicy 2014	184 (2)
5% weight loss	Cercato 2009, Suplicy 2014	184 (2)
Discontinuation due to adverse reactions	Cercato 2009, Suplicy 2014	184 (2)
Change in waist circumference	Cercato 2009, Suplicy 2014	184 (2)
Change in body weight	Cercato 2009, Suplicy 2014	184 (2)

Network diagrams: short-term treatments

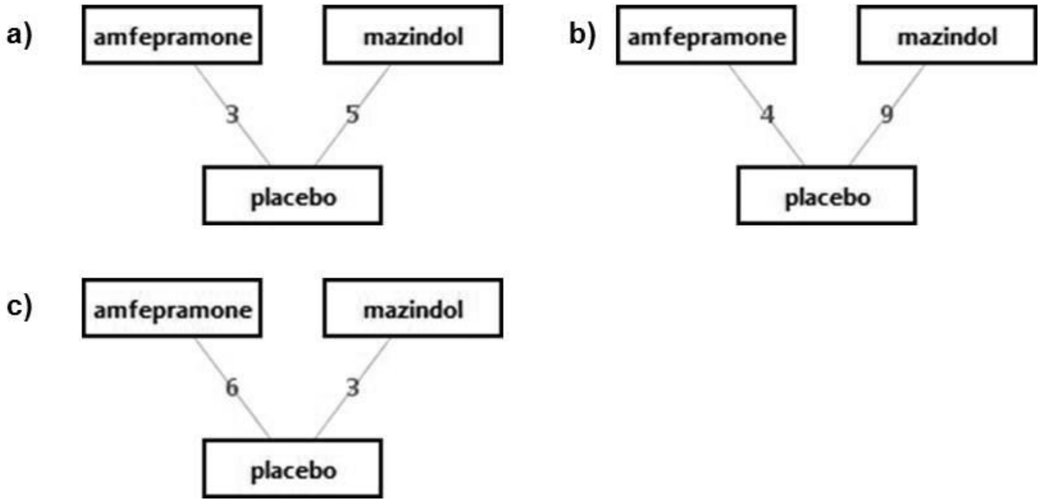

The lines represent the direct comparisons. a) Change in body weight, b) discontinuation due to adverse drug reactions, and c) participants with at least one adverse reaction.

Network diagrams: short-term treatments

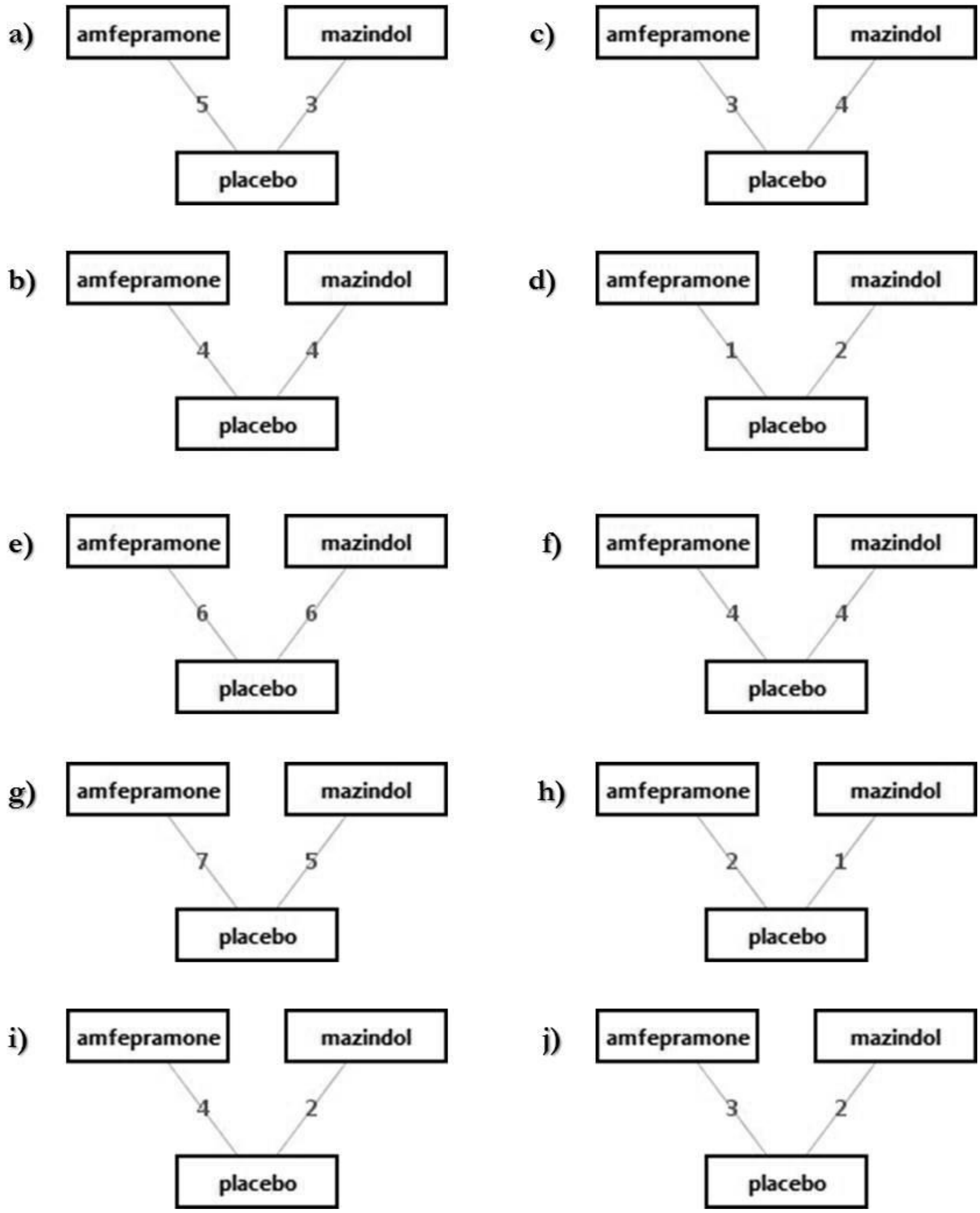

The lines represent the direct comparisons. a) Nausea, b) dry mouth, c) vomiting, d) constipation, e) dizziness, f) insomnia, g) headache, h) somnolence, i) tension, and j) irritation.

Network diagram: long-term treatments

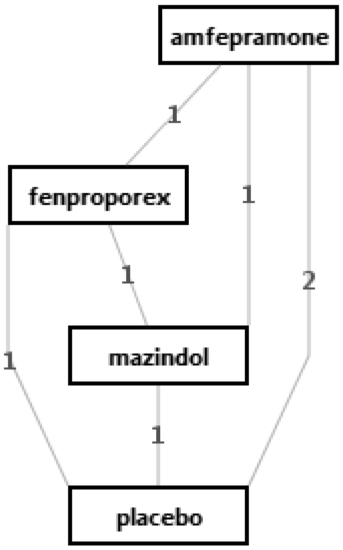

The lines represent the direct comparisons. Outcomes: change in body weight, change in waist circumference, 5% weight loss, 10% weight loss, discontinuation due to adverse reactions.
